# Artificial radionuclides in the plant cover around nuclear fuel cycle facilities

**DOI:** 10.1371/journal.pone.0306531

**Published:** 2024-07-02

**Authors:** Natalya Larionova, Anna Toporova, Pavel Krivitskiy, Vasiliy Polevik, Natalya Lechshenko, Valeriy Monayenko, Mariya Abisheva, Viktor Baklanov, Assan Aidarkhanov, Vladimir Vityuk

**Affiliations:** 1 Branch ‘Institute of Radiation Safety ad Ecology’ RSE NNC RK, Kurchatov, Kazakhstan; 2 Shakarim State University, Semey, Kazakhstan; 3 Branch ‘Institute of Atomic Energy’ RSE NNC RK, Kurchatov, Kazakhstan; 4 National Nuclear Center of the Republic of Kazakhstan, Kurchatov, Kazakhstan; University of South Carolina, UNITED STATES

## Abstract

This paper presents research on the assessment of the radioecological state of plant cover surrounding two research reactor facilities located within the Semipalatinsk Test Site (STS) as examples of nuclear fuel cycle facilities (NFC). Source data on the concentrations of artificial radionuclides in the plant cover were obtained. Quantitative values for ^137^Cs, ^241^Am, and ^239+240^Pu activity concentrations were determined in plants across the perimeters of the facilities, indicating that these compounds may be present in the associated media from the perspective of accumulative bioindication. The values determined for artificial radionuclides in the ‘soil‒plant’ system around the researched NFC facilities were attributed to radioactive contamination of the STS territory.

## 1. Introduction

Research dedicated to studying the accumulation of artificial radionuclides by plants has been the most extensive since the beginning of nuclear energy utilisation. At that time, ^60^Co, ^90^Sr, ^137^Cs, ^3^H, and Pu isotopes were released into the environment at different stages of the nuclear fuel cycle because of both normal and off-normal operation of atomic power facilities (radiation accidents and incidents). Radioecological research dedicated to this issue is mentioned in literature for the 30-km impact zone of the Beloyarsk nuclear power plant (NPP) [1], region of four NPPs in Sweden [2], NPP ‘Kozloduy’ in Bulgaria [3], Ignalina NPP northeast of Lithuania [4], Kaiga NPP in India [5,6], and others.

Nuclear accidents and incidents have also significantly contributed to the contamination of natural ecosystems. Research on the migration parameters of artificial radionuclides in the land cover of natural pastures contaminated by accidents (1986) at the Chernobyl NPP [7] was conducted in Belarus, Russia, Ukraine [8,9], Great Britain [10,11], Italy [12,13], and Sweden [14]. The most recent large accident occurred in ‘Fukushima-1’, Japan (2011). To date, Japanese scientists have conducted a series of studies on the radionuclide accumulation by plants growing in the contaminated zones [15]. The ‘Fukushima-1’ accident also led to radioactive contamination of the environment in the far east of Russia. The maximum concentrations of ^134^Cs and ^137^Cs in plants (on a fresh weight basis) on the Sakhalin and Kuril Islands in 2011 were 5 and 18 Bq/kg, respectively [16]. The results of environmental radioactivity studies and assessments of radiation doses to humans and biota were recently summarised in the comprehensive review book entitled “Fukushima Accident: 10 years after” [17].

Most of the aforementioned research has been dedicated to determining the migration of ^137^Cs and ^90^Sr from the soil to plants. However, at some stages of the nuclear fuel cycle (NFC) at the fuel and energy facility, especially at the stage related to the reprocessing of irradiated fuel, the release of transuranic radionuclides into the environment–plutonium isotopes, in particular, ^239+240^Pu–may be possible. Fewer studies have studied accumulation of ^239+240^Pu in plants [18–20] than that of ^137^Cs and ^90^Sr.

Radioactive contamination produced by nuclear testing is characterised by the presence of high concentrations of transuranic radionuclides (^241^Am, ^239+240^Pu) in environmental compartments [21–25]. Much research on the assessment of ^137^Cs, ^90^Sr, ^241^Am, and ^239+240^Pu concentrations [26–28] in plant cover has been conducted at the Semipalatinsk Test Site (STS). Research on the parameters of radionuclide accumulation by plants, points to the cumulative capacity of plant cover and individual plant species to varying degrees. Plants can thus be used as accumulative bioindication when monitoring NFC facilities.

**Fig 1:**
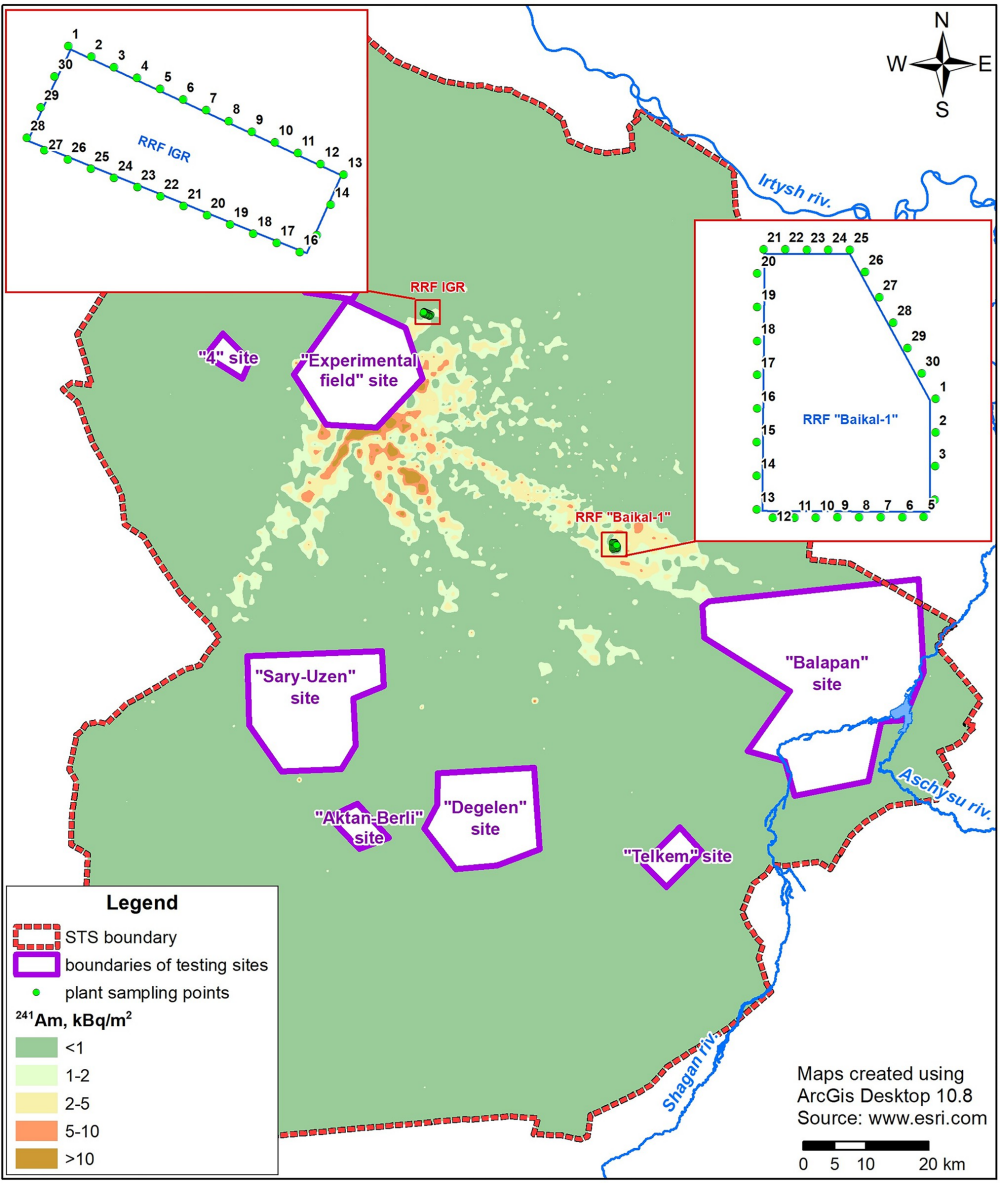
Map of ^241^Am areal activity distribution in STS soil cover [40], at locations of research objects and sampling points.

NFC facilities are located in the STS territory and include two research reactor facilities (RRF IGR and Baikal-1). The RRF IGR was commissioned in 1960 and was designed for nuclear testing of fuel elements and assemblies, as well as experimentation to justify safety at nuclear engineering facilities. The IGR reactor is a pulse graphite reactor on thermal neutrons with a homogeneous core representing a pile of uranium-containing graphite blocks assembled in the form of columns. The RRF Baikal-1 was commissioned in 1975 and was designed for testing fuel elements and assemblies as well as for researching reactor materials and safety rationale experimentation. Fig 1 shows the locations of the research objects in the STS.

For decades, within the framework of scientific and technical programs dedicated to the development of nuclear energy, numerous studies have been conducted in areas of immediate problems related to the safety of nuclear and fusion reactors [29], material science problems [30,31] thermonuclear research [32], and areas related to radioecology and safety issues. The existing monitoring of environmental pollution around objects RRF, IGR, and Baikal-1, which is a prerequisite for their use, reflects the situation at a given time in most cases, but is not always able to provide an integral estimate for the previous period. From this point of view, this paper offers a promising object of observation: plants, which may act as biological monitoring sites that characterise the radiation situation over a certain period of time.

## 2. Materials and methods

The plant cover area of interest was studied using separate geobotanical techniques with major vegetation types and species composition of the identified plants [33]. The radiation parameters, β-particle fluence, and equivalent dose rate (EDR), which are necessary for primarily assessing the presence of any radioactive contamination in the area of interest, were measured during the field work according to standard procedures [34].

The sampling points were located using the ArcGIS software system for Desktops. The perimeters of the objects of interest were demarcated from satellite images. Equally spaced point coordinates (~30 m) were used in this study. Each object was scored 30 points (Fig 1).

To determine the concentrations of radionuclides in the plants, plant samples were collected thrice at each point in the summertime (in July) for two years. ^137^Cs, ^90^Sr, ^241^Am, and ^239+240^Pu were selected as the controllable artificial radionuclides of interest. To assess the radionuclide content in the associated media, the soil was simultaneously sampled at 10 points (as deep as 0–5 cm) chosen from the analytical results of plant samples.

Field work under this Agreement was carried out by the "Institute of Radiation Safety and Ecology," a branch of the RSE NNC RK based on State License No. 19014221 dated 07/03/2019 for the provision of services in the field of atomic energy use, issued by the RSE "NNC RK" State Institution "Committee of Atomic and energy supervision and control of the Republic of Kazakhstan".

Laboratory activities included general soil and plant sample preparation for analysing and determining the content of radionuclides of interest. The plant samples were coarsely crushed (1–3 cm long), flushed and rinsed 2–3 times in distilled water, and then dried in a beaker at 80–100°C. A laboratory mill was used for fine crushing. The samples were then thermally concentrated (charred and ashed). The dry residue was charred in a muffle furnace by calcinating the electrolytes without sample inflammation, until a black precipitate was produced. The samples were then cooled, ground, transferred to porcelain cups or crucibles, followed by ashing. Initially, the temperature was increased to 200°C for 50–60 minutes, after which the following temperature limits were set in the muffle furnace: the ashing temperature to determine ^137^Сs was 400°С, and the temperature for ^90^Sr, ^241^Am, and ^239+240^Pu was 500°С. The resulting ash was sieved to remove ash-free residue. Soil samples were air-dried in beakers at 60–70°С, mixed, gradually (in portions) ground in a porcelain mortar and sieved through a 1 mm mesh. Radionuclide activity concentrations in soil and plant samples were analytically measured as per standardised guidelines [35,36] using calibrated equipment. The activity concentrations of ^137^Cs and ^241^Am were determined using a Canberra GX-2020 gamma-spectrometer, and those of ^90^Sr and ^239+240^Pu were determined by radiochemical isolation followed by a TRI-CARB 2900 TR beta-spectrometer and a Canberra alpha-spectrometer (mod. 7401, respectively). The concentrations of ^137^Cs, ^241^Am, ^90^Sr, and ^239+240^Pu in the plants were determined in the ash, followed by conversion to dry weight (d.w.). The average coefficient of ashing was 0.34±0.05 (400°С), and the average coefficient of ashing was 0.060±0.004 (500°С). The minimum detectable activities (MDA) for ^137^Cs were 0.2 Bq/kg (for plant samples) and 2.0 Bq/kg (for soil samples), those for ^90^Sr were 0.5 Bq/kg and 1 Bq/kg, and those for ^241^Am were 0.1 Bq/kg and 0.5 Bq/kg, and those for ^239+240^Pu were 0.1 Bq/kg and 1 Bq/kg.

The radionuclide determinations were subjected to quality checks. One test sample and one ‘blank’ sample were added to each batch of the analysed samples (10 samples per batch). The test sample was randomly selected from the set of samples included in the batch, whereas the ‘blank’ sample was prepared in advance using samples collected from territories with ‘background’ contents of technogenic radionuclides. The test and ‘blank’ samples were analysed simultaneously with the remaining samples. The test sample was intended to control the quality and repeatability of the analytical results, whereas the ‘blank’ sample was used to control hypothetical sample cross-contamination.

Maps quoted in the paper were constructed using ArcGIS software based on digitised maps of the Republic of Kazakhstan that were acquired by the branch ‘Institute of Radiation Safety and Ecology’ NNC RK from the Republican Public State Enterprise the ‘National Mapping and Geodesic Fund’ of the Committee for Geodesy and Mapping. The Ministry of Digital Development, Innovations, and Aerospace Industry of the Republic of Kazakhstan under the State Procurement Agreement No. 02-19/122 dated 2020-04-28.

## 3. Results and discussion

Based on the geobotanical description data, the plant cover in the area of interest was found to be dry xerophytic-motley-sod grass steppes on light-chestnut soil. The vegetation was represented by a complex of gramineous-sandy needle-grass-absinthial associations: sarepta feather grass (*Stipa sareptana*), esparto grass (*Stipa capillata*), sheep fescue (*Festuca valesiaca*), Lessing’s feather grass (*Stipa lessingiana*), June grass (*Koeleria cristata*), thin sagebrush (*Artemisia gracilescens*), Marshall sagebrush (*Artemisia marschalliana*) and others. The average projective cover was 60–80%. The terrain was represented by low mountains, hummock plains, and plain regions of desert steppes. The dominant species of the sagebrush genus (*Artemisia gracilescens* and *A*. *marschalliana*) were selected as the plant species of interest.

Measurements of radiometric parameters revealed that the β-particle fluence in the area of interest was <0.10 p/(cm^2^ × min), and the equivalent gamma dose rate on the soil surface on average varied from 0.10–0.14 μSv/hour.

The contents of ^137^Cs and ^241^Am in the plant cover across the perimeter of the RRF IGR are below the detection limit of the methodological instrumentation used. An exception was two points with numerical values for the content of ^137^Cs (0.6±0.1 (No. 5) and 0.5±0.1 Bq/kg (No. 6)). Table 1 presents the results of the gamma-spectrometric analysis of the plant samples collected in the vicinity of RRF Baikal-1 in June of the first year.

**Table 1:** Results of activity concentrations for artificial ^137^Cs and ^241^Am in plants.

Study area in the vicinity of RRF "Baikal-1"							
Point No.	Activity concentration in plants (d.w.), Bq/kg		Dry mass, kg	Point No.	Activity concentration in plants (d.w), Bq/kg		Dry mass, kg
	^137^Cs	^241^Am			^137^Cs	^241^Am	
1	0.8±0.3	0.8±0.2	0.079	16	1.3±0.3	0.6±0.1	0.074
2	0.8±0.2	0.5±0.1	0.106	17	-	-	0.075
3	1.6±0.3	0.6±0.1	0.118	18	-	-	0.096
4	1.7±0.3	0.6±0.1	0.090	19	1.1±0.2	0.6±0.1	0.101
5	0.9±0.2	3.9±0.8	0.128	20	0.7±0.1	0.6±0.1	0.084
6	-*	1.0±0.2	0.105	21	-	0.3±0.1	0.086
7	-	2.5±0.5	0.109	22	-	-	0.065
8	0.6±0.1	-	0.096	23	0.5±0.1	-	0.081
9	-	-	0.076	24	-	-	0.066
10	-	1.2±0.2	0.107	25	0.7±0.3	0.4±0.1	0.067
11	0.6±0.1	3.5±0.7	0.054	26	0.4±0.1	-	0.053
12	0.8±0.2	-	0.084	27	0.7±0.2	-	0.090
13	0.8±0.2	-	0.111	28	0.4±0.1	-	0.141
14	1.5±0.3	0.6±0.1	0.072	29	-	-	0.076
15	-	-	0.080	30	1.3±0.3	0.9±0.2	0.126

-*–below MDA.

The activity concentrations of artificial ^137^Cs and ^241^Am in the plant cover in the vicinity of RRF Baikal-1 varied from <0.2 to 1.7±0.3 Bq/kg and <0.1 to 0.9±0.2 Bq/kg, respectively.

In most cases, quantification of gamma-emitting radionuclide concentrations in plants in the vicinity of the RRF IGR failed. Thus, further research into the content of ^137^Cs and ^241^Am in plants was undertaken in the second year; moreover, ^90^Sr and ^239+240^Pu were determined in the vicinity of RRF Baikal-1.

Tables 2 and 3 provide the results of the analysis of ^137^Cs, ^90^Sr, ^241^Am, and ^239+240^Pu in plants and soils simultaneously sampled in the second year at 10 points, with elevated concentrations of ^137^Cs and ^241^Am in the plant samples (Table 1).

**Table 2:** Results of the activity concentrations of ^137^Cs and ^90^Sr in plants and soil in the vicinity of RRF Baikal-1.

Point No.	Activity concentration, Bq/kg				Transfer factors (Tf)	
	^137^Cs		^90^Sr		^137^Cs	^90^Sr
	plants (d.w.)	soil	plants (d.w.)	soil		
3	0.3±0.1	3.1±0.6	-	-	0.10	-
4	-*	12±2	-	1.5±0.4	-	-
5	0.4±0.1	21±4	-	2.4±0.4	0.02	-
7	-	8.1±1.6	-	-	-	-
11	0.3±0.1	19±4	-	1.4±0.5	0.02	-
14	0.6±0.1	19±4	-	1.1±0.4	0.03	-
16	-	9.6±1.9	-	-	-	-
19	0.4±0.1	16±3	-	1.0±0.5	0.03	-
25	-	4.9±1.0	-	-	-	-
30	1.6±0.3	43±8	-	2.4±0.4	0.04	-

*–below MDA.

**Table 3:** Results of the activity concentrations of ^241^Am and ^239+240^Pu in plants and soil in the vicinity of RRF Baikal-1.

Point No.	Activity concentration, Bq/kg				Transfer factors (Tf)	
	^241^Am		^239+240^Pu		^241^Am	^239+240^Pu
	plants (d.w.)	soil	plants (d.w.)	soil		
3	-*	0.6±0.1	-	-	-	-
4	-	6.0±1.2	-	131±21	-	-
5	-	8.9±1.8	0.3±0.1	134±15	-	0.0022
7	-	6.5±1.3	-	70±10	-	-
11	-	9.3±1.9	0.3±0.1	128±18	-	0.0023
14	-	6.2±1.2	-	44±7	-	-
16	-	2.7±0.5	-	41±8	-	-
19	-	6.8±1.4	-	47±11	-	-
25	-	3.3±0.6	-	22±9	-	-
30	1.3±0.3	33±6	0.5±0.1	375±40	0.04	0.0013

*–below MDA.

**Fig 2:**
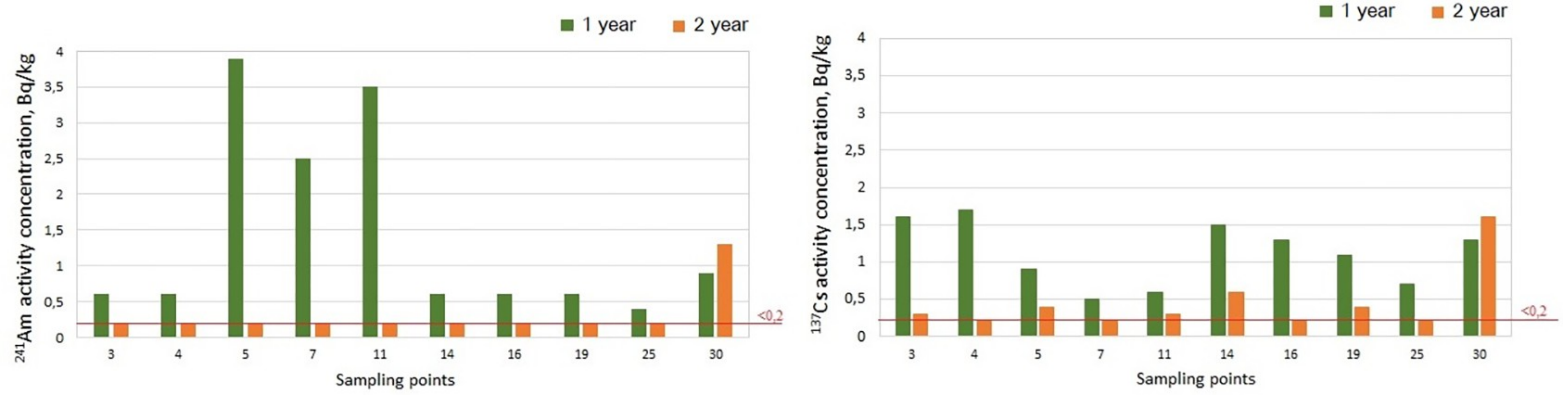
Content of ^241^Am (а.) and ^137^Cs (b.) in the plant cover in the vicinity of RRF Baikal-1 in the first and second research years.

Based on the analytical results, the ^90^Sr activity concentration in the soil of this territory did not exceed 2.4±0.4 Bq/kg, and that in the plants was below the detection limit of the methodological instrumentation used. The elevated values of ^137^Cs (to 43 Bq/kg) and ^241^Am (33 Bq/kg) as well as the high values of ^239+240^Pu (to 375 Bq/kg) in soil indicate that in this case, it may act as a source of these radionuclides entry into plants. This is proven by the fact that with an elevated concentration of a radionuclide in soil, corresponding quantitative values are also always registered in plants. For instance, the ^137^Cs quantitative values for plants were determined at points No. 5, 11, 14, 19 and 30, with an elevated content of this radionuclide in soil (>16 Bq/kg), except at point 3 (3.1 Bq/kg). The ^239+240^Pu concentrations in plants were determined at points 5, 11, and 30, where the content of this radionuclide in the soil was increased (>130 Bq/kg), with the exception of point 4. The only quantitative value of ^241^Am concentration in the plants was recorded at point 30, where the activity concentration of this radionuclide in soil was 33 Bq/kg. Simultaneously, this point was characterised by the highest radioactive contamination with all radionuclides, and quantitative values of ^137^Cs and ^241^Am were detected in plants both in the first and second research years (Fig 2).

Generally, in the second research year, a reduced content of both radionuclides in plants was noted (Fig 2), which may have been related to lower precipitation in that year. The annual amount of precipitation in the first research year was 198 mm, and that in the second year was 162 mm [37]. Simultaneously, Cs+ root uptake decreases with decreasing soil moisture [38]. The ^137^Cs activity concentrations varied from <0.3 to 1.6±0.3 Bq/kg in the second research year, and those of ^241^Am were below the detection limit of the methodological instrumentation (except at point 30). Under these conditions, this finding may point to a certain concentration threshold for radionuclides in the soil, below which the accumulative properties of plants as indicators are invalid (considering the methodological instrumentation used). Based on the findings for ^137^Cs, such a threshold is limited to approximately 16±3 Bq/kg, below which the concentration of this radionuclide in plants is not always registered. For ^241^Am and ^239+240^Pu, in this case, this value is somewhat greater (33±6 Bq/kg and 128 Bq/kg, respectively), which is attributable to their lower transfer factor (Tf) [26,27,39].

Tfs were calculated as the ratio of the activity concentration of a radionuclide in plants to that in soil–for a more detailed evaluation of the parameters of radionuclide accumulation by plants from soil as a possible contamination source. Only the absolute ’real’ values were used to estimate Tf. The Tf values of ^137^Cs ranged from 0.04 to 0.1 (Table 2), those of ^239+240^Pu ranged from 0.0013 to 0.0023, and those of ^241^Am—0.04 (Table 3).

It was previously established that the geometric mean (GM) was suitable for expressing the mean Tf values for radionuclides [26]. The mean Tf values determined for ^137^Cs, ^239+240^Pu, and ^241^Am, are compiled in Table 4. For comparison, Table 4 provides the Tf previously derived for other STS areas, which are defined by a similar contamination type: epicentres of aboveground tests, “plumes” of radioactive fallout–the territory of radioactive contamination produced by radioactive particles that settled on the terrain from the cloud of a nuclear (a zone of local (not global) fallout), and conventionally “background” STS areas–the territory with the content of artificial radionuclides in topsoil at the level of global fallout between the former testing areas and “рlumes” of radioactive fallout [26]. A comparative analysis was also carried out with summarised international Tf values (IAEA, 2009) for pasture grass collected from loamy soils (also characteristic of STS territory) [39].

**Table 4:** Geometric mean ^137^Cs, ^241^Am, and ^239+240^Pu Tf values in the researched STS territories [26] and International generalised data [39].

Territories	Mean values of Tf of radionuclides		
	^137^Cs	^241^Am	^239+240^Pu
Research territory	0.034 (6)*	0.04 (1)	0.0019 (3)
earlier researched territories STS			
epicentres of aboveground testing	0.0028 (72)	0.00052 (73)	0.0014 (66)
“plumes” of radioactive fallout	0.020 (30)	0.0056 (23)	0.0068 (29)
conventionally “background” areas	0.030 (40)	-	0.019 (33)
tecdoc (IAEA.2009)			
pasture	0.2 (124)	0.001 (11)	0.0003 (10)

in brackets–number of observations.

The Tf values of ^137^Cs derived for the researched area are significantly higher (by an average order of magnitude) than those determined for the epicentres of aboveground nuclear tests and are the closest to those derived earlier for “plumes” of radioactive fallout and for conventionally “background” areas STS. The Tf values for ^137^Cs derived from the territory of interest were one order of magnitude lower, and those of ^239+240^Pu and ^241^Am were higher than the Tf values for these radionuclides in the pasture (Table 4).

In general, no trend toward an elevated or reduced accumulation of radionuclides by the plants in the study area was found. Thus, the non-standard entry of radionuclides into the land cover of this territory, for example, into a soluble form in water or aerosols, is currently excluded. Therefore, the influence of the nuclear fuel cycle facilities (research reactor facilities RRF-IGR and Baikal-1) on the surrounding area has not been fixed. The presence of values determined for artificial radionuclides in the ‘soil‒plant’ system can be attributed to radioactive contamination of the STS territory [40]. First of all, this is the fallout of radioisotopes from the atmosphere as a result of an explosions at the "Experimental field" site (Fig 1).

## Conclusion

Data on the concentrations of artificial radionuclides ^137^Cs, ^90^Sr, ^241^Am, and ^239+240^Pu in the plant cover around the two research reactor facilities located at the STS were obtained. The contents of ^137^Cs and ^241^Am in the plant cover across the perimeter of the RRF IGR were below the detection limit of the methodological instrumentation used. The activity concentrations of artificial ^137^Cs, ^241^Am, and ^239+240^Pu in the plant cover in the vicinity of RRF Baikal-1 vary from <0.2 to 1.7±0.3 Bq/kg, from <0.1 to 0.9±0.2 Bq/kg, and from <0.1 to 0.5±0.1 Bq/kg, respectively, and the quantitative values of ^90^Sr concentration in plants remain to be established. The dynamics of the contents of artificial ^241^Am and ^137^Cs were established by a reduction in plant cover, which was revealed in the second research year. The quantitative values determined for artificial radionuclide activity concentrations in plants across the perimeter of the facilities, overall, suggest the presence of radionuclides in associated media from the perspective of accumulative bioindication. However, the presence of values determined for artificial radionuclides in the ‘soil‒plant’ system around the researched nuclear fuel cycle facilities was attributed to radioactive contamination of the STS territory.

## References

[1] Molchanova I.V., Karavayeva, Ye N., Kulikov N.V., 1985. Some outcomes of the radioecological study of natural ecosystems in the zone of the Beloyarsk NPP. Ecology. 5, 30–34.

[2] Mascanzoni D., 1989. Plant uptake of activation and fission products in a long-term field study. J. Environ. Radioact. 10 (3), 233–249. 10.1016/0265-931X(89)90027-1.

[3] Djingova R., Kuleff I., 2002. Concentration of cesium-137, cobalt-60 and potassium-40 in some wild and edible plants around the nuclear power plant in Bulgaria. J. Environ. Radioact. 59 (1), 61–73. 10.1016/S0265-931X(01)00036-4. 11848152

[4] Lukšienė B., Marčiulionienė D., Gudelienė I., Schönhofer F., 2013. Accumulation and transfer of ^137^Cs and ^90^Sr in the plants of the forest ecosystem near the Ignalina Nuclear Power Plant. J. Environ. Radioact. 116, 1–9. 10.1016/j.jenvrad.2012.09.005. 23085187

[5] Joshy, James P., Dileep B. N., Ravi P. M., Raghunandan, Joshi M., Ajith T. L., Hegde A. G., et al., 2011. Soil to leaf transfer factor for the radionuclides ^226^Ra, ^40^K, ^137^Cs and ^90^Sr at Kaiga region, India. J. Environ. Radioact. 102 (12), 1070–1077. 21868141 10.1016/j.jenvrad.2011.07.011

[6] Karunakara N., Ujwal P., Yashodhara I., Rao Chetan, Sudeep Kumara K., Dileep B. N., et al., 2013. Studies on soil to grass transfer factor (Fv) and grass to milk transfer coefficient (Fm) for cesium in Kaiga region. J. Environ. Radioact. 124, 101–112. doi: 10.1016/j.jenvrad.2013.03.008 23685702

[7] Israel Yu. A., 1990. Chernobyl: radioactive contamination of natural environments. Leningrad: Gidrometeoizdat, 295 p. (in Russian).

[8] Shutov V.N., Bekyasheva Т.А., Basalayeva L.N., 1993. The influence of soil properties on the entry of ^137^Cs and ^90^Sr into natural motley grasses. Soil sceince. 8, 67–71.

[9] Askbrant S., Sandalls J., 1998. Root uptake of ^137^Cs and ^90^Sr by rye-grass on various soils in the CIS. J. Environ. Radioact. 38 (1), 85–95. 10.1016/S0265-931X(97)00017-9.

[10] Sandalls J., Bennett L., 1993. Radiocaesium in upland herbage in Cumbria, UK: A three year field study. J. Environ. Radioact. 19 (3).147–165.

[11] Rudge S.A., Johnson M.S., Leah R.T., Jones S.R., 1993. Biological transport of radiocaesium in a semi-natural grassland ecosystem. Soils, vegetation and invertebrates. J. Environ. Radioact. 19 (3), 173–198. 10.1016/0265-931X(93)90002-O.

[12] Lotfi M., Notaro M., Azimi-Garakani D., Cubadda R., Santaroni G.P., Tommasino L., 1990. Concentrations of radiocaesium in Italian durum wheat and its products after the Chernobyl accident. J. Environ. Radioact. 11 (2), 177–182. 10.1016/0265-931X(90)90060-9.

[13] Velasco R.H., Toso J.P., Belli M., Sansone U., 1997. Radiocesium in the Northeastern part of Italy after the chernobyl accident: Vertical soil transport and soil-to-plant transfer. J. Environ. Radioact. 37 (1), 73–83. 10.1016/S0265-931X(96)00078-1.

[14] Fircks Y., Rosen K., 2002. Uptake and distribution of ^137^Cs and ^90^Sr in Salix viminalis plants. J. Environ. Radioact. 63 (1), 1–14. 10.1016/S0265-931X(01)00131-X. 12230132

[15] Tagami K., Uchida S., Ishii N., Kagiya Sh., 2012. Translocation of radiocesium from stems and leaves of plants and the effect on radiocesium concentrations in newly emerged plant tissues. J. Environ. Radioact. 111, 65–69. doi: 10.1016/j.jenvrad.2011.09.017 22027214

[16] Ramzaev V., Barkovsky A., Goncharova Yu, Gromov A., Kaduka M., Romanovich I., 2013. Radiocesium fallout in the grasslands on Sakhalin, Kunashir and Shikotan Islands due to Fukushima accident: the radioactive contamination of soil and plants in 2011. J. Environ. Radioact. 118, 128–142. doi: 10.1016/j.jenvrad.2012.12.006 23344426

[17] Povinec P.P., Hirose K., Aoyama M., Tateda Y., 2021. Fukushima Accident: 10 Years after (second ed.), Elsevier, New York (2021).

[18] Salminen-Paatero S., Nygren U., Paatero J., 2012. 240Pu/239Pu mass ratio in environmental samples in Finland. J. Environ. Radioact. 113, 163–170. doi: 10.1016/j.jenvrad.2012.06.005 22776691

[19] Edomskaya M. A., Lukashenko S. N., Shupik A. A., Korovin S. V., Tomson A. V., 2022. Assessment of plutonium isotopes content in soil at the vicinity of the radioactive waste storage facility in Obninsk. Radiation and Risk. 31 (4), 73–81. 10.21870/0131-3878-2022-31-4-73-81.

[20] Luo M., Liu D., Dai X., Wu Y., Ni Y., 2023. Determination of ultra-low level of 239,240Pu in grass/vegetable sample by compact accelerator mass spectrometry. J. Radioanal. Nucl. Chem. 10.1007/s10967-022-08753-9.

[21] Kashirsky V.V., Shatrov A.N., Zvereva I.O., Lukashenko S.N., 2020. Development of a method for studying 241Pu/241Am activity ratio in the soil of the main Semipalatinsk test site areas. J. Environ. Radioact. 216 (4), 106181. 10.1016/j.jenvrad.2020.106181. 32056789

[22] Krivitskiy P. Ye., Larionova N. V., Baklanova Yu. V., Aidarkhanov А. O., Lukashenko S. N., 2022a. Characterization of area radioactive contamination of near-surface soil at the Sary-Uzen site in the Semipalatinsk test site. J. Environ. Radioact. 249 (2–3), 106893. doi: 10.1016/j.jenvrad.2022.106893 35569206

[23] Krivitskiy P.Ye, Larionova N.V., Monayenko V.N., Subbotin S.B., Chernov A.A., Panitskiy A.V., 2022b. Peculiarities of radioactive soil contamination in places of underground nuclear tests in the Semipalatinsk test site. J. Environ. Radioact. 253–254, 106991. doi: 10.1016/j.jenvrad.2022.106991 36084569

[24] Kunduzbayeva A.Ye., Lukashenko S.N., Kabdyrakova A.M., Larionova N.V., Magasheva R.Yu., Bakirova G.A., 2022. Speciation of ^137^Cs, ^90^Sr, ^241^Am, and ^239+240^Pu artificial radionuclides in soils at the Semipalatinsk test site. J. Environ. Radioact. 249 (10), 106867. 10.1016/j.jenvrad.2022.106867. 35523044

[25] Aidarkhanova A., Larionova N., Tleukanova Zh., Mamyrbaeva А., Ermakova R., Svetacheva Yu, et al., 2022. The character of radionuclide contamination of natural lakes at the territory of the Semipalatinsk test site. J. Environ. Radioact. 255, 107041. doi: 10.1016/j.jenvrad.2022.107041 36265400

[26] Larionova N.V., Lukashenko S.N., Kabdyrakova А.М., Kunduzbayeva A.Y., Panitskiy A.V., Ivanova A.R., 2018. Transfer of radionuclides to plants of natural ecosystems at the Semipalatinsk Test Site. J. Environ. Radioact. 186, 163–170. doi: 10.1016/j.jenvrad.2017.09.006 28992995

[27] Larionova N.V., Lukashenko S.N., Lyakhova O.N., Aidarkhanova А.К., Kunduzbayeva A.Ye., Kabdyrakova A.M., et al., 2021. Transfer parameters of radionuclides from soil to plants at the area of craters produced by underground nuclear explosions at the Semipalatinsk test site. J. Environ. Radioact. 237 (1–2), 106684. doi: 10.1016/j.jenvrad.2021.106684 34186240

[28] Kozhakhanov T.E., Lukashenko S.N., Larionova N.V., 2014. Accumulation of artificial radionuclides in agricultural plants in the area used for surface nuclear tests. J. Environ. Radioact. 137, 217–226. doi: 10.1016/j.jenvrad.2014.06.026 25128979

[29] Gordienko Y., Ponkratov Y., Kulsartov T., Tazhibayeva I., Zaurbekova Z., Koyanbayev Y., et al. Research Facilities of IAE NNC RK (Kurchatov) for Investigations of Tritium Interaction with Structural Materials of Fusion Reactors.—Fusion Science and Technology. - №6.—Vol. 76. - 2020.—P. 703–709. 10.1080/15361055.2020.1777667.

[30] Kulsartov T.V., Udartsev S.V., Samarkhanov K.K., Gordienko Yu.N, Ponkratov Yu.V., Baklanova Yu.Yu., et al. The temperature-time dependence of the amount and type of niobium beryllides formed during the synthesis of the binary intermetallic compound NbBe3. Intermetallics. - 2023.—Vol. 163, 108065. 10.1016/j.intermet.2023.108065.

[31] Skakov M., Baklanov V., Kukushkin I., Toleubekov K., Bekmuldin M., Akaev A., et al. Investigation of the interaction between corium and metal-coolers at the VCG-135 test bench in the conditions of a severe accident. Nuclear Engineering and Design, 2024, 424, 113296. 10.1016/j.nucengdes.2024.113296.

[32] Kashaykin P.F., Tomashuk A.L., Vasiliev S.A., Ignatyev A.D., Shaimerdenov A.A., Ponkratov Y.V., et al. Radiation resistance of single-mode optical fibres with view to in-reactor applications.—Nuclear Materials and Energy.—Vol. 27. - 2021. - №. 100981. 10.1016/j.nme.2021.100981.

[33] Lavrenko, E. M., Korchagin, A. A., 1959–1976. Field geobotany. Moskva. 1–5. (in Russian).

[34] ICRCNE USSR, 1989. Instructions and guidelines on the ground survey of the radiological situation in the contaminated area. Goskomgidromet. Moskov. (in Russian).

[35] MP 5.06.001.98 RK "Activity of radionuclides in bulk samples. The MP 2143–91 was measured with a gamma spectrometer.18 p.

[36] A procedure to determine isotopes of plutonium (-239+240), strontium-90, americium-241 in environmental sites, 1998. Almaty. MP 06-7-98.

[37] Weather and climate, 2004. Reference portal. http://www.pogodaiklimat.ru/history/36152.htm (accessed on April 10, 2023).

[38] Nikitin A.N., 2021. Impact of soil moisture on cesium uptake by plants: Model assessment. J. Environ. Radioact. 240, 106754. doi: 10.1016/j.jenvrad.2021.106754 34607179

[39] IAEA, 2009. Quantification of Radionuclide Transfer in Terrestrial and Freshwater Environments for Radiological Assessments, 2009. Vienna. https://www.iaea.org/publications/8103/quantification of radionuclide-transfer-in-terrestrial-and-freshwater-environments-for-radiological-assessments.

[40] Batyrbekov, E., Aidarkhanov, A. Vityuk V., Larionova, N., Umarov, M., Comprehensive radioecological survey of Semipalatinsk Test Site, 2021. Kurchatov. 339 p.

